# Impact of exon 19 versus exon 21 EGFR-activating mutation on outcomes with upfront pemetrexed–carboplatin chemotherapy

**DOI:** 10.3332/ecancer.2017.776

**Published:** 2017-10-24

**Authors:** Vanita Noronha, Vijay Patil, Amit Joshi, Anuradha Chougule, Atanu Bhattacharjee, Rajiv Kumar, Sucheta More, Supriya Goud, Ashay Karpe, Anant Ramaswamy, Nikhil Pande, Arun Chandrasekharan, Alok Goel, Vikas Talreja, Abhishek Mahajan, Amit Janu, Nilendu Purandare, Kumar Prabhash

**Affiliations:** 1 Department of Medical Oncology, Tata Memorial Hospital, HBNI, Parel, Mumbai 400068, India; 2 Department of Biometrics, Chiltern International Limited, Residency Road, Bengaluru 560025, India; 3 Department of Pathology, Tata Memorial Hospital, HBNI, Parel, Mumbai 400068, India; 4 Department of Radiology, Tata Memorial Hospital, HBNI, Parel, Mumbai 400068, India; 5 Department of Nuclear Medicine, Tata Memorial Hospital, HBNI, Parel, Mumbai 400068, India; * These authors contributed equally to this work.

**Keywords:** EGFR mutation, NSCLC, exon 19, pemetrexed, exon 21, gefitinib, prognosis

## Abstract

**Background:**

EGFR mutation subtype is a recognised factor impacting outcomes of patients receiving oral tyrosine kinase inhibitors (TKIs) in non-small-cell lung cancer (NSCLC). Evidence for the effect of this factor on outcomes in patients receiving pemetrexed is limited.

**Methods:**

We completed a study comparing pemetrexed–platinum combination versus oral TKI in EGFR mutation-positive patients in lung cancer. We analysed the impact of EGFR mutation subtype, specifically, exon 19 and 21 on the PFS and OS of patients treated with pemetrexed (500 mg/m^2^ on day 1) and carboplatin (AUC 5 on day 1) as first-line therapy. Patients underwent axial imaging for response assessment on D42, D84, D126 and subsequently every two months till progression. Patients post-progression were treated with gefitinib.

**Results:**

Fifty-one patients (36%) had exon 21 mutation, while 92 patients (64%) had exon 19 mutation. Response rates in evaluable patients was 47.7% in exon 19 patients (41 patients, *n* = 86) and 42.9 % in exon 21 patients (18 patients, *n* = 42). There was a significant increase in median overall survival for patients with exon 19 mutations (24.5 months, 95% CI: 21.3–27.7 months ) over the exon 21-mutated patients (18.1 months, 95% Cl: 13.5–22.6 months, *p* = 0.002). This differential impact was due to second-line gefitinib having a differential outcome on these mutations.

**Conclusion:**

Pemetrexed-based chemotherapy does not have a differential impact on exon 19- or exon 21-mutated patients. However, second-line treatment with gefitinib has a favourable response and outcome in exon 19-mutated patients.

## Introduction

The heterogeneity in EGFR mutations in terms of biology and potential response to treatment has been a work in progress from the time of the discovery of these mutations in NSCLC [1]. Deletion in exon 19 and L858R substitution on exon 21 constitute a majority of these mutations and they predict for a high response rate to tyrosine kinase inhibitors. These mutations were termed as classic activating mutations [2, 3].

Multiple studies have established that upfront tyrosine kinase inhibitors (reversible or irreversible) are the treatment of choice in patients with classic activating mutations. However, these studies have clubbed exon 19 deletions and exon 21 L858R mutations together [4–6]. There is growing evidence to suggest that exon 19 deletion may have a better prognosis compared to exon 21 mutations [7]. A recent meta-analysis of over 2500 patients with classic activating mutations reported that patients with exon 19 deletions have better progression-free survival compared to exon 21 L858R mutations, when treated with irreversible TKIs [8]. Also, exon 19 deleted patients treated with irreversible TKI (afatinib) had an overall survival advantage according to the LUX-LUNG studies [9]. In the near future, it is likely that these mutations may need to be addressed separately when conducting studies.

However, not uncommonly, patients with EGFR mutations are treated with pemetrexed platinum chemotherapy. It is unknown whether the response, progression-free survival and overall survival of these patients are influenced by the type of EGFR mutation. A *post hoc* analysis of a phase-III trial was carried out to address this question.

## Methods

### Study

We completed a single-centre, phase-3, double arm, parallel-group, open-label exploratory randomised study comparing pemetrexed with carboplatin and oral TKI in EGFR mutation positive lung cancer patients (Clinical trial registry of India: CTRI/2015/08/006113). We have reported its results [10]. This was a *post hoc* analysis planned with a primary objective to compare the progression-free survival between exon 19 deleted and exon 21L858R-mutated cohort when treated with pemetrexed carboplatin. The secondary objective was to compare the response rate and overall survival.

### Patient selection

The detailed eligibility criteria of the original study are published elsewhere [10] For this analysis, we selected patients subjected to the following selection criteria. We included adult (age > or = 18 years) patients with ECOG PS 0-2, with either exon 19 deletion or exon 21L858R mutation, with pathologically confirmed adenocarcinoma without uncontrolled comorbidities, with adequate organ function and receiving first-line treatment with pemetrexed–carboplatin chemotherapy. Patients who had received upfront gefitinib or who had exon 18 mutation were excluded from this analysis.

### Intervention

Patients were treated with six cycles of pemetrexed (500 mg/m^2^) and carboplatin (AUC-5) with appropriate antiemetics and supportive care at three weekly intervals. Post-six cycles, patients who had non-progressive disease were offered pemetrexed maintenance. The chemotherapy was continued till development of progressive disease or intolerable side effects or any other protocol defined criteria.

Patients underwent response assessment scan after three cycles, six cycles and then at two monthly intervals. At progression, all patients were offered gefitinib and were followed up till death.

### Statistical analysis

SPSS version 20 was used for analysis. Response rate at the end of third cycle was documented in accordance with RECIST version 1.1 and compared with Fisher’s exact test. Progression-free survival was defined as time in months from randomisation to objective PD (progressive disease), change in treatment or death from any cause. Patients who had not progressed at last follow up were censored on 14th July 2016. Overall survival was defined as time in months from randomisation to death from any cause. Patients who had not died at last follow-up were censored on 14th July 2016. Kaplan–Meier time to event analysis was carried out for the estimation of PFS and OS. Log rank test was used for the comparison of PFS and OS between exon 19 deleted and exon 21–mutated patients. The COX regression analysis was used to estimate the hazard ratio with its 95% confidence interval. A *p* value of 0.05 or below was considered as significant.

## Results

### Baseline details

A total of 143 patients received pemetrexed-based therapy as first-line treatment for stage-III/stage-IV NSCLC in the chemotherapy arm. 51 patients (36%) had exon 21 mutation, while 92 patients (64%) had exon 19 mutation. The median age was 54 years (27–74 years). There were 95 males (66.4%) and 48 females (33.6%). Twenty-eight patients (19.6%) had a history of previous smoking. The stage was stage IIIB in three patients and stage-IV disease in the remaining patients. The ECOG PS was 0–1 in 129 patients (90.2%) and two in 14 patients (9.8%). The distribution of baseline characteristics in accordance with the type of mutation is shown in Table 1. The ECOG PS was slightly imbalanced between the two cohorts with 98.0% having ECOG PS 0–1 status in exon 21 mutated cohort versus 93.5% in exon 19 cohort. Imbalance was also observed in incidence of brain metastasis at presentation; 9.8% in exon 21 cohort as against 19.6% in exon 19 deletion cohort.

### Response rate

Out of 143 patients, 15 patients were ineligible for response assessment. The overall response rate among evaluable patients was 46.1% without any complete responses (Table 2). Response rates in evaluable patients was 47.7% in exon 19 patients (41 patients, *n* = 86) and 42.9 % in exon 21 patients (18 patients, *n* = 42) (*p* = 0.706, Fisher’s exact two-sided *p* value).

### PFS

**Figure 1.**
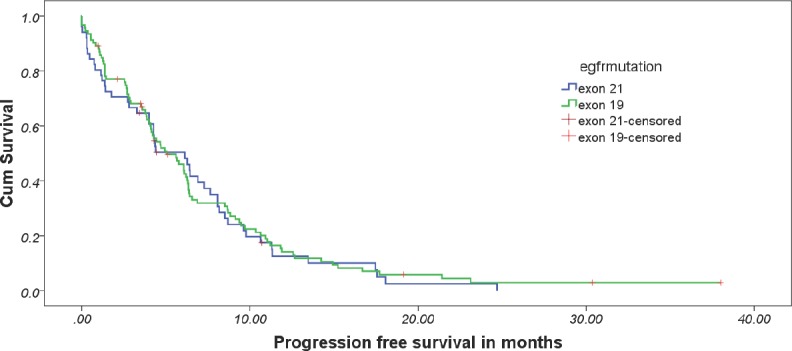
Estimated progression-free survival between the two cohorts.

At the data cut-off, 90.6% of the patients had progressed. The overall median PFS was 5.033 months (95% CI: 3.596–6.471). The median PFS in exon 19 and 21 cohorts were 5.033 months (95% CI: 3.428–6.638) and 6.133 months (95% CI: 3.815–8.452), respectively (Figure 1). There was no differential impact of EGFR mutation on PFS (*p* = 0.599, HR = 0.904, 95% CI: 0.620–1.317). Table 3 provides details of cox regression analysis results.

### OS

**Figure 2.**
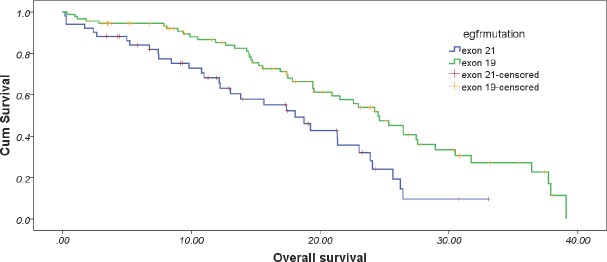
Estimated overall survival between the two cohorts.

At the data cut-off, 65.2% of the patients had died. The overall median survival was 21.533 months (95%CI: 17.558–25.508 months). The median overall survival in exon 19 patients was 24.5 months (95% CI: 21.3–27.7 months ) which was significantly better than that seen in exon 21 mutated patients (18.1 months, 95% Cl: 13.5–22.6 months, *p* = 0.002) (Table 4 and Figure 2).

### Impact of second-line gefitinib

Out of 128 patients who had progressed, 16 patients had not received gefitinib. The second-line treatment with gefitinib was received in 112 patients, of which 76 patients were in the exon 19 cohort and 36 in the exon 21 cohort. Ninety-eight patients were evaluable for response and the response rate was 69.7% in the exon 19 cohort as opposed to 43.8% in the exon 21 cohort. The median progression-free survival was 8.067 months (95% CI: 6.556–9.577 months) in exon 19 cohort versus 5.767 months (95%CI: 3.814–7.719 months) in exon 21 cohort (*p* = 0.141). The median overall survival was 17.867 months (95%CI: 13.079–22.655 months) in the exon 19 cohort as opposed to 9.633 months (95% CI: 5.959–13.308 months) in the exon 21 cohort (*p* = 0.001). The patients who had not received gefitinib had poor survival and the median OS was only 9.4 months versus 22.6 months in patients receiving it (*p* = 0.012).

### Discussion

Biomarkers are frequently used in oncology and certain biomarkers like activating EGFR mutations have both prognostic and predictive implications [2]. Both exon 19 deletions and exon 21 L858R substitutions have been considered together when landmark trials were done and reported in first line setting [4–6]. Recent data suggest that both these mutations have different biological behaviours and have different prognostic significance when treated with reversible and irreversible tyrosine kinase inhibitors like gefitinib, erlotinib or afatinib [7–9]. However, it is not known how tumours carrying these mutations would behave if they were exposed to chemotherapy. The current analysis was done to answer this question.

We failed to identify any major difference in baseline characteristics or tumour metastasis pattern between the cohorts carrying the two mutations. The response rate to chemotherapy and median PFS was similar between the two groups indicating that pemetrexed carboplatin therapy with pemetrexed maintenance had a similar impact on both groups irrespective of the type of EGFR mutation. Similar findings were reported in an ASCO 2015 annual conference abstract by Kogure *et al.* [11]. In their study of 32 patients, they reported response rates of 33.3% with exon 21 L858R substitution versus 22.2 % with exon 19 deletion, a median PFS of 5.13 months with exon 21 L858R substitution versus 5.40 with exon 19 deletion and a similar OS.

In our study, the median OS was different between the two groups favouring the exon 19 cohort with a hazard ratio of 0.425. The improvement in OS without any improvement in PFS indicated influence of therapy provided post progression on first-line treatment. Hence, the *post hoc* analysis of the influence of type of EGFR mutation on response to second-line therapy, second-line PFS and second-line OS was performed. The outcomes were similar to those reported in the literature for first-line setting. Exon 19 deleted patients had a better response rate, a better median PFS and a better median OS in comparison to exon 21-mutated cohort. This implies that the differential behaviour of exon 19 and 21 mutations is seen in the presence of tyrosine kinase inhibitors and not with chemotherapy irrespective whether the exposure to TKI was in the first-line setting or second-line setting.

The current analysis has its own limitations. It was a single-centre study, *post hoc* analysis with a small sample size and the cohorts were not balanced for important prognostic factors like the presence or absence of brain metastasis and ECOG PS.

## Conclusions

Pemetrexed-based chemotherapy does not have a differential impact on exon 19 or exon 21-mutated patients. However, second-line treatment with gefitinib has a favourable response and outcome in exon 19-mutated patients.

## Figures and Tables

**Table 1.** Baseline characteristics between the two cohorts.

Variable	Exon 19 (*n* = 92)	Exon 21 (*n* = 51)
Median age	53 (30–73)	55 (27–74)
Gender Male Female	60 (65.2%) 32 (34.8%)	35 (68.6%) 16 (31.4%)
ECOG PS 0–1 2	86 (93.5%) 6 (6.5%)	50 (98.0%) 1(1.0%)
Habits Ex smoker	18 (19.6%)	10 (19.6%)
Stage III IV	2 (2.2%) 90 (97.8%)	1 (2.0%) 50 (98.0%)
Brain metastasis	18 (19.6%)	05 (9.8%)
Liver metastasis	26 (28.3%)	14 (27.5%)

**Table 2.** Response to pemetrexed between the two cohorts.

Variable	Exon 19 (*n* = 92)	Exon 21 (*n* = 43)
CR (complete response)	-	-
PR (partial response)	41	18
SD (stable disease)	33	20
PD (Progressive disease)	12	4
Not evaluable	6	9

**Table 3.** Details of multivariate analysis for progression-free survival.

Variable	Hazard ratio	*p* value on Cox regression analysis
Elderly	0.635 (0.387–1.040)	0.071
Gender	1.517 (1.032–2.229)	0.034
ECOG PS	1.252 (0.556–2.818)	0.587
Smoking status	1.140 (0.717–1.814)	0.580
Liver metastasis	1.499 (1.012–2.220)	0.043
Brain metastasis	1.357 (0.836–2.201)	0.216
EGFR mutation type	0.904 (0.620–1.317)	0.599

**Table 4.** Details of multivariate analysis for overall survival.

Variable	Hazard ratio	*p* value on Cox regression analysis
Elderly	0.586 (0.308–1.117)	0.105
Gender	1.671 (0.999–2.796)	0.051
ECOG PS	1.063 (0.358–3.154)	0.912
Smoking status	1.145 (0.636–2.059)	0.652
Liver metastasis	1.284 (0.777–2.119)	0.329
Brain metastasis	2.312 (1.262–4.236)	0.007
EGFR mutation type	0.425 (0.255–0.709)	0.001
